# A qualitative study of health experiences of Ethiopian asylum seekers in Norway

**DOI:** 10.1186/s12913-019-4813-7

**Published:** 2019-12-11

**Authors:** Yvette Louise Schein, Brita Askeland Winje, Sonja Lynn Myhre, Ingunn Nordstoga, Melanie Lindsay Straiton

**Affiliations:** 1 Perelman School of Medicine at the University of Pennsylvania, Philadelphia, PA USA; 2 Department of Vaccine Preventable Diseases, Norwegian Institute of Public Health, Oslo, Norway; 3 Department of International Public Health, Norwegian Institute of Public Health, Oslo, Norway; 4 LHL’s International Tuberculosis Foundation, Oslo, Norway; 5 Department of Mental Health and Suicide, Norwegian Institute of Public Health, Oslo, Norway

**Keywords:** Refugees, Asylum seekers, Resettlement, Healthcare experiences, Barriers to care, Mental health

## Abstract

**Background:**

Norway, like other European countries, has a growing refugee population. Upon arrival to Norway, refugees and asylum seekers need to learn about Norwegian society and social services such as healthcare. Despite various programs and assistance, they face numerous challenges using the healthcare system. Understanding the healthcare experiences of Ethiopian refugees and asylum seekers may improve how services such as informational sessions and delivery of medical care are provided. This qualitative study seeks to describe the health-related experiences of Ethiopians who have sought asylum in Norway and shed light on potential barriers to care.

**Methods:**

Individual interviews were conducted with ten Ethiopian refugees and asylum seekers in Norway. Thematic analysis was used to understand the broader context of refugee resettlement and how this experience influences participants’ health experiences and health seeking behaviors.

**Results:**

We identified three main themes that played a role in participants’ health and healthcare experiences. Participants described how ‘living in limbo’ during their application for residency took a mental toll, the difficulties they had ‘using the healthcare system’, and the role ‘interpersonal factors’ had on their experiences. While applying for asylum, participants felt consumed by the process and were affected by the lack of structure in their lives, the conditions in the reception center, and perceived inadequate healthcare. Participants perceived a change in access to services before and after they had been granted residency. Participants learned about the healthcare system both through official information sessions and social networks. Doctor-patient communication and interpersonal factors such as a sense of feeling valued, language, and discrimination had a large impact on perceived quality of care.

**Conclusions:**

Ethiopian refugees and asylum seekers face numerous challenges accessing, using, and interacting with Norway’s healthcare system. Contextualizing these challenges within the asylum seeking process may help policy makers better understand, and therefore address, these challenges. Interventions offered at reception centers and in health worker trainings may improve healthcare experiences for this and similar populations.

## Background

The number of people displaced due to conflicts, natural disasters, and human rights violations has increased in recent years. The United Nations High Commissioner for Refugees (UNHCR) estimates that at the end of 2017, there were approximately 71.44 million such persons of concern globally [1]. This estimate refers in part to refugees, those granted protection by another state, and asylum seekers, those who have sought international protection but whose refugee status has not yet been determined [1].

While Iran, Lebanon, Pakistan, Uganda, and Turkey bear the greatest burden of displaced people [2], Europe also has a history of accepting refugees and asylum seekers. In 2017, over 25,000 refugees were resettled in Europe by the UNHCR. The top countries for resettlement included the UK, France, Sweden, the Netherlands, Germany and Norway [3]. As of June 2018, there were 228,161 people with refugee backgrounds living in Norway [4]. Asylum seekers face numerous challenges accessing and utilizing health services and may have very different healthcare experiences from the native population [5]. Given the large number of people with refugee background living in Norway, understanding how challenges using the healthcare system manifest themselves is an important research and national policy priority. This study, using a sample of Ethiopian refugees and asylum seekers, aims to understand the healthcare experiences of this community and elucidate important factors that may be generalizable to other refugee groups and host countries regarding barriers and facilitators of care and utilization of health services.

### Healthcare experiences and barriers to care

In many of the countries where refugees to Europe originate, the healthcare system is fragmented and inefficient [6], and organized differently than in Europe. Ethiopia is among 57 countries identified by the WHO to have a critical shortage of healthcare workers [7]. Furthermore, with regards to quality of care, one Ethiopian study reported that Ethiopian patients and health care providers found an inadequate number and mix of professional staff, a deficiency of basic medical equipment, and lack of compassion and respect for patients among health care providers, all of which may influence their experience with other health care systems [8]. Even the idea of scheduling an appointment may be unfamiliar to some, thus increasing unnecessary utilization of emergency departments. Scandinavian studies using local register data find that immigrants tend to use emergency departments for all health problems more than natives, although this varies by country background and immigration reason [9–12].

Language and cultural background are also key mediators of healthcare experiences. A study of language barriers in the United States found that patients who face language barriers are less likely to have a regular source of medical care, have increased risk of non-adherence to prescribed medication, and are less likely to return for follow up appointments after visits to the emergency room [13]. Similarly, a Canadian study reported that length of stay in emergency departments was longer for non-English speakers compared to English speakers [14]. Language barriers were also found to be an important obstacle to care in Norway [15].

Differences in cultural background between physician and patient have been shown to create an environment of mistrust in some cases, both in terms of confidentiality and expertise. A study of Somali refugee expectations of healthcare in Sweden found that unfulfilled expectations of medical encounters can result in disappointment that entails “a lack of trust and feelings of rejection.” [16] Some studies have also found that perceived differential treatment on the basis of immigrant status has negative effects on health seeking behavior [15, 17–19]. Many Norwegian healthcare providers have also expressed frustration regarding challenges in providing care to recently arrived migrants [20].

### Introduction program for new refugees and asylum seekers

New asylum seekers to Norway live in reception centers located throughout the country. They and their families are required to participate in a standardized introduction program [21]. The program has been in place since 2004 and is organized as a collaboration between the Norwegian Welfare and Administration (NAV), adult education centers, and volunteer organizations and businesses [22]. The aim of the program is for participants to learn Norwegian, obtain knowledge about Norwegian society (including the healthcare system) and become prepared to enter the labor market or pursue further education [23].

### Norway’s healthcare system and healthcare rights for asylum seekers

Norway’s healthcare system is a universal, publicly funded system. Those who qualify for this system are assigned a general practitioner (GP) who acts as a gatekeeper for referrals to specialty care and hospital services. Residents may change their GP up to twice a year if they desire [24]. Co-payments for office visits and other costs are subsidized by the government, and all costs exceeding 2205 NOK (260 USD) per year are covered by the national healthcare system [25].

According to the Norwegian Directorate of Immigration (UDI), those seeking asylum have the same rights to healthcare as Norwegian citizens from the moment they apply for protection in Norway [26]. It is only in the case of denied asylum and undocumented stay in Norway that immigrants do not have complete access to the health system [27, 28]. Additionally, upon arrival in Norway, asylum seekers are required to undergo tuberculosis screening, and should be offered HIV testing and full healthcare screening including dental and mental health. Asylum seekers under the age of 20 are also entitled to a full vaccination program [29].

Despite full legal access to the healthcare system, asylum seekers to Norway still face numerous challenges accessing and utilizing the health services, especially in the transit phase [30]. With this in mind, this study aims to describe the health related experiences of Ethiopian refugees and asylum seekers in Norway and discuss the barriers and facilitators of access to care in this community.

## Methods

### Interview procedure

We set out to interview 30 participants, or until data saturation was reached. We identified participants through key contacts in the Ethiopian community such as interpreters and church administrators. Contacts served as recruiters and identified and contacted potential participants. We then used snowball sampling to recruit additional participants [31]. Selection criteria included those who were originally from Ethiopia, arrived in Norway as refugees or asylum seekers, were 18–65 years old, had been living in Norway between six months and 10 years, and had visited a doctor while in Norway.

Once participants agreed to be interviewed, they were contacted by telephone by the first author, and a suitable time and place were arranged for a meeting. Since our community recruiters had the first interaction with the majority of potential participants, we do not know how many people declined to be contacted for an interview.

At the time of the interview, participants were given an information sheet and consent form, both of which were available in English and Amharic. All interviews took place between February and June 2017. The study was approved by the Data Protection Officer and conducted in accordance with the Norwegian Data Protection Authority.

The primary goal of the interview was to learn about participants’ healthcare experiences in Norway. We used a semi-structured interview guide (Additional file 1 – Interview Guide) with open-ended questions relating to differences in the health system between Norway and Ethiopia, experiences of going to the doctor and making appointments, and barriers to care such as cost, transportation, sociocultural differences, language, and trust. At the completion of each interview, participants received a 200 NOK (~ 23.70 USD) gift card to compensate them for their time. The first author, a native English speaker, interviewed all participants. A professional interpreter was available for those not proficient in English, and three participants used this service. All interviews were audio-recorded and transcribed. Interview transcripts ranged in length from 12 to 30 pages (mean 19.1).

### Participants

The final study sample consisted of 10 Ethiopians who entered Norway as asylum seekers. Participants were between 20 and 54 years old (mean 30.3 years); half were female, and length of time in Norway ranged from three to ten years (mean 6.8 years). Eight participants were from Addis Ababa, the capital of Ethiopia, while two were from more rural areas. At the time of the interview, seven participants were permanent residents of Norway, one had been granted citizenship, and two had not yet received full residency. Five participants entered Norway as minors and were therefore granted immediate access to education. Four participants were university students studying to obtain a bachelor’s degree; one participant was a high school student; two were neither in school nor working (high school education obtained in Ethiopia); and three were working full time. Of those working, one had an advanced degree. Eight participants were living in Oslo at the time of the interview, while two lived in neighboring towns. At the time of the interview, six participants assessed their health as very good, three as good, and one as fair.

### Analysis

To analyze the interviews, we employed thematic analysis, which aims to identify patterns and themes within the recorded text without trying to fit data into pre-existing themes [32]. By taking an inductive approach, we were able to consider the broader context of refugee resettlement and how this experience influences participants’ health experiences and health seeking behaviors. Additionally, this method allowed us to identify the major themes of barriers to and facilitators of care and to consider how asylum seekers have adapted to using the health care system.

The research team reviewed the transcripts and the first author coded units into analytic categories. Each category was then reviewed for cohesion and compared to coding done by the last author for consistency. By comparing and contrasting categories across the interviews and referring back to the full interviews, the categories were grouped into higher order themes. NVivo 11 was used to assist in coding and analyzing transcripts. All interviews were de-identified to protect participants. Participant names were only used for contact purposes and were not connected to the interview data in any way.

## Results

Refugees and asylum seekers experience a wide array of challenges that influence healthcare utilization and experiences. Their experiences varied by time in Norway and residency status. The period of uncertainty that accompanies the asylum seeking process consumes the lives of asylum seekers and makes it difficult to be conscious of health status or seek care. Those who are granted residency then need to learn to navigate healthcare services with limited assistance. Finally, those who learn how the system works are faced with complex social interactions in the clinician’s office that can significantly influence health seeking behavior.

### Living in limbo

Participants spoke profusely about the mental toll the asylum seeking process had on them. Long term uncertainty about the future often causes stress and anxiety. For many it was an all-consuming and isolating experience that made it difficult to feel happy and fulfilled.

#### Uncertainty

The asylum application process can take many years. Some participants reported waiting eight or nine years to be granted asylum, and this constant worry about residency status was reported as having direct health implications. One participant noted, “*it’s not so easy to live in a refugee camp. So I was worried if I was going to get a permit or not so it was a stressful time for me. So I was not sleeping very well so I went to a doctor and it was not that serious, but I got some help for that*” (R7, F).

The asylum seeking process was perceived as overwhelmingly stressful, and this was compounded by the limited ability to pursue useful work activity. “*When a person is sitting in a camp without doing anything, that person will be stressed and so will be sitting and thinking about what will happen in the future”* (R4, M). Uncertainty and not having work, responsibility, or diversions results in even greater stress levels.

In addition to its impact on mental and physical health, the resettlement process also had direct consequences that limited health seeking behavior. One participant stated “*I think maybe I don’t know I wasn’t focused on my health … I was focused on my situation rather than my health”* (R1, F). Another, when asked about expectations for the healthcare system in Norway stated, “*you know what … when I came to Norway it wasn’t the first thing that I thought about. Because I had so many things … other things to think about. So I didn’t think [about it] so much”* (R2, M).

#### Lack of structure

The limitations of living in a resettlement center, too, were a large source of unhappiness for participants. One participant described being unable to access education because it was too far from the asylum center and having to find her own living accommodations through Facebook to make it work. Others described being unable to work and the uselessness they felt as a result. One participant in particular spoke a lot about what this lack of work meant for his mental health: “*My health is good but when one is living in a camp most of the time life is very stressful … it is difficult to live with so many people. In a very closed area and also this is the age where I should be working or going to school and I am not doing anything but sitting the whole day here”* (R4, M). Another participant, when asked what he thought could be done to improve health services for asylum seekers stated,

*Everything I wish could happen is so many people who are sitting doing nothing in the camp … I wish if Norwegian people or government can help them to get to allow them to get a job... Everyone has lived by working and doing something in their lives.... If you sit all the time, all the year, 2 years, 3 years, in one camp … you will be mad. You will be sick.* (R9, F)

#### Conditions at the reception center

Many of the reception centers that participants were placed in were in small towns or rural locations far from cities. One participant stated that she preferred her town in the north of Norway to Oslo because “[I] *found friends that were [my] age and then school was so easy because there was only one class for each year. So it was easier to make friends”* (R1, F). Another found the location of the camp he was living in to be incredibly isolating: *“there was nothing around it. There was only forest, nobody there, so all you do most of the time is sit. So after I have moved it has become easier for me to come to town, to go to church, and yeah it was better. It was closer to town”* (R4, M). Moving to a more populated area greatly improved this participant’s mental status.

Conditions at the reception centers also had an impact on participants’ physical health. Participants spoke of friends with special needs, such as diabetes and hypertension, who were unable to modify their diets in the reception centers to meet their health needs: *“for example, this man must not eat sugary things for his diabetes and for his blood pressure he must not eat salty things. But they [the reception center] will give you [them anyway]*” (R10, M). This participant explained how the reception center initially refused to prepare a special menu for one person. This person, however, knew the importance of glycemic control, and refused to leave the doctor’s office at his next visit until the doctor called the reception center to advocate for healthier food options. Thus, in order to maintain good health in the reception center, asylum seekers have to both be aware of how to control their health conditions and how to advocate for adequate care.

### Using the healthcare system

Once asylum seekers can mentally move past the magnitude of their situation, they must also learn how to use a healthcare system that is completely different from their prior experiences. Gaining refugee status after a long period of waiting appears to influence the perception of access to health services. Participants spoke about the ways in which they learned about the healthcare system, differences in access related to residency status, and understanding the role of the GP.

#### Perceived limited access prior to gaining residency

Every respondent spoke about the differences they or their friends experienced between having or not having residency “papers”. Prior to gaining residency, participants described doctors coming to the reception centers occasionally or when someone was very sick. Doctors seem to have been in high demand at this stage, and “*when once in a while when the doctor would come to the reception center and check everybody and that time everybody would go and see him*” (R1, F). Similarly, respondents described having to get a letter from the reception center in order to see a doctor outside the center, making it difficult to access health services in the local community. In this scenario, participants preferred the Ethiopian healthcare system where “*the doctors really want to help you … and everybody can get the treatment if they got the money. But over here, people who are not like legal residents cannot get the treatment we are getting”* (R6, F). Thus, despite the fact that asylum seekers and refugees have legal access to healthcare, the perception by this participant is that treatment is not available.

Participants also struggled with finding a doctor, changing doctors, and with feelings of discomfort due to perceived lower quality of care than that received by Norwegian citizens. One participant stated that: *“when you don’t have a paper it’s not easy to find the doctor. You just have nurses … and when you have the papers then everything is ok. Everything is working or they will help you to find your own doctor and everything when you find this number … person number they call it … but when you don’t have that number it’s so difficult”* (R1, F). The personal number, Norway’s national identification number, allows one to be identified in the system and is the way to be processed through the healthcare service. Without this number, participants described a sense of invisibility: “*you are invisible and nobody cares about you … they don’t count us. Then [once you get your permit] you are visible*” (R6, F). Although asylum seekers are assigned temporary personal numbers upon arrival in Norway, this information may not be clearly communicated, and clearly affects perceptions of inclusiveness and health seeking behavior.

While living in a reception center, the kind of health professionals also varies. While doctors are only at most reception centers just a few days a week, nurses are there every day. Some participants mentioned frustration at having to see a nurse when a doctor was not available and subsequently feeling as if they had not received adequate care: “*it is hard to go to the nurse and talk about all my illness or when I know they actually can’t help me that much”* (R3, M). These experiences in the reception center determine the mindset with which new asylum seekers approach learning about and utilizing their new healthcare system.

#### Perceived universal access after gaining residency

Once granted residency, participants perceived that their healthcare access was then the same as Norwegian citizens. This access was discussed primarily in the context of cost. *“[I am} very satisfied with their system. When somebody gets sick, one does not pay that much it’s just a little bit so it’s almost free... The rest is paid by the government”* (R7, F). This was in stark contrast to the health system in Ethiopia: “*in Ethiopia if you have money if you can afford it they will respect you. But if you have no money there is no respect. If you don’t have money you don’t get treatment in Ethiopia”* (R8, F).

Those who expressed negative experiences regarding cost spoke about the cost of some specialty services, such as optometry and dentistry, which are not covered for adults in the healthcare system in Norway. Additionally, when asked if they had ever had difficulty paying for an appointment, participants responded that they were given a month to pay a bill (often at a price of around 200 NOK - 24 USD) or that the costs were completely covered by NAV. One participant, whenever given a bill for health services, stated that she *“applies to NAV and they usually provide the payment for it”* (R8, F). In this population, cost is not a barrier to accessing healthcare services.

#### Information about healthcare services

The vast majority of participants remembered extensive information sessions provided by the reception center, UDI, or NAV. One participant described, *“they will give you documents. They are saying what to do when you are sick, when it’s a fire at home, which number you’re going to call when it’s an emergency and just like that”* (R5, M). While nearly all reported receiving this information, a similar majority reported being unable to use this information because they were too young to see it as important, it was too much information at once, or it was too much information too early in their asylum seeking process. One respondent reported that *“we learned a lot but it was really boring just sitting there and trying to listen. All the unnecessary stuff we actually thought we wouldn’t have any use at the time since we were like oh we’ve never used that. You don’t think like that when you’re 15”* (R3, M). This statement suggests that the information was poorly contextualized in regards to the differences between what asylum seekers could expect in Norway in comparison to what they may have previously experienced. Though information was communicated, the information was not necessarily transferable into health seeking skills.

Despite information sessions, many participants were wholly reliant on social services to mediate healthcare appointments and were unable to act autonomously. Participants spoke about relying on a contact person at NAV to facilitate all healthcare interactions in the first years of their stay in Norway: *“in the beginning it was very difficult, but now I know how to do it. I can go to the clinic and make an appointment … in the beginning I didn’t know where to go, who to ask, who to talk to, so they helped me in the beginning”* (R8, F).

A handful of the participants stated that their family was their primary source of information when learning about the Norwegian healthcare system. Others used friends to find a good specialist or, when asked what he looks for in a doctor, one participant stated, *“I may ask my friends which one is good and yeah their experiences. So when they tell me ok he is a better one so you should take that one so then I may choose that guy”* (R2, M). Informal tips passed through social networks were important in navigating day-to-day issues with the healthcare system.

#### Role of the general practitioner

Participants described going to the doctor in Ethiopia only when they were acutely ill. The preexisting lack of a notion of preventive care affected their perception of health care in general, as well as how or when to access it. Most participants were not used to making appointments at all. In Ethiopia, they would go to the doctor when sick and wait their turn in line to be seen. These prior experiences are important to consider in trying to understand this community’s healthcare experiences and their use of Norway’s GP-mediated system. One participant acknowledged this cultural divide in health seeking behavior: *“yeah if you have that kind of situation [preventive care] if you are practicing in your own country you can do it here. But if you don’t have that practice like that then you wouldn’t”* (R10, M).

Learning how to optimize one’s interaction with an assigned GP, to change a GP when necessary, and to make appointments are essential parts of using Norway’s healthcare system. The majority of participants knew how to change doctors, and the majority had in fact used that knowledge. One respondent reported going through eight doctors before finding a satisfying doctor: “*Every year I changed the doctors because I was not at all satisfied by the way they treated their patients. Like yeah it’s hard sometimes to find the nice doctors. … I went through eight doctors...* (R3, M). There were, however, some participants who did not know how to change their doctor.

Participants also commented on the difficulty of making appointments. One participant stated, *“if it’s very horrible like if I get sick and I have to see the doctor right way I can’t they give me an appointment but it’s like … it’s like I need to see the doctor right away right … but they give me an appointment and I don’t like that”* (R2, M). The majority of participants who expressed frustration with making appointments had difficulty with wait times. Respondents described Norway’s healthcare system as one where “*You don’t get immediate help when you are sick … it takes a long time”* (R4, M). Navigating this timing was a challenge to many participants. One stated that making an appointment was *“so much process”* (R1, F). Such statements imply a hesitancy to deal with the appointment making process. This may be due to being accustomed to the healthcare system in Ethiopia where appointments are unnecessary. However, the other half of participants reported no difficulty seeing primary care providers: *“no problem. Even now if I am sick if I go if it is a working day, you can get it”* (R10, M).

In addition to waiting for an appointment, participants also discussed frustration waiting to see a doctor once already in the office:

*they gave me an appointment and I showed up by the date and time. But after 3 hours or something waiting they said, no we can’t do it today because the doctor is busy. Or something. And they gave me another appointment for after a week … it was disappointing. Yeah. They told you to come today and you need to wait like 3 hours and someone may show up and tell you to go back home.* (R6, F)

Such experiences can be a deterrent to seeking care in the future.

Frustrations with wait times were exaggerated when trying to access specialty care. Participants described the process of accessing a specialty physician as challenging, and were surprised to learn that they might have to wait up to a year to see a particular doctor. Participants were split regarding their attitudes towards Norway’s referral system of care. Half felt that it was better: *“it is better that I be referred by my regular doctor or primary doctor because he knows about my problem or my health situation and he can assess it better than me”* (R8, F), while the other half would have preferred more direct access. While the goal of gatekeeping systems is to promote efficient use of healthcare services, and is the shared experience of all Norwegians, it may also limit access to subspecialty care disproportionately for those who are unfamiliar with this system.

### Interpersonal factors and the doctor-patient relationship

Doctor-patient relationships and communication are complicated by many factors including language, trust or mistrust, and sociocultural differences. Participants found this aspect of their healthcare experiences particularly challenging.

#### Being valued

It was important to participants that their doctors explain everything fully. Such full and detailed explanations fostered trust. Similarly, participants spoke about the importance of doctors *“speaking really closely”* (R6, F) with them. In such circumstances, participants found their doctors to be the most helpful and were able to form a therapeutic alliance. Participants felt they were taken seriously if their doctors had been thorough and had checked any possible cause of their complaint.

*I have some problems with migraines and I have been with the doctors so many times but over the years all of them just say just take one day free and you will be fine tomorrow and until one doctor actually took his time and tried to understand why I am having the unexplained migraines till then no one just … everyone just said like take water or take painkillers and take it easy and you will be fine. So it’s just like I think I changed six doctors since I was not satisfied with each doctor because nobody just took their time to see that and actually try to understand why I am having these headaches.* (R3, M)

Participants felt more satisfied with their health interactions when the doctor *“actually physically checks on you and tries to understand [the problem] and try to take time … and actually seeing from each problem”* (R3, M).

Participants also felt strongly that it was important for their doctors to show a personal interest in their care. One participant, whose resettlement process was particularly difficult because her husband was not granted asylum, spoke positively about her relationship with her doctor who had written to UDI (The Norwegian Directorate of Immigration) on her and her husband’s behalf many times. Other participants discussed what it meant to them that even when they had nothing more to talk to the doctor about specifically, the doctor always made a point to ask them about their day. These small gestures, in combination with satisfactory healthcare outcomes, convinced participants that they were being adequately cared for.

A majority of participants described an experience of going to the doctor for a perceived health issue and being told to “just drink water.” Participants were disheartened by this kind of advice: *“water is good for health, we know. But for every sickness it’s not helpful. They say go and drink water, go and drink water”* (R10, M). While it may be that there was nothing to do from a medical standpoint for a given complaint, such statements from physicians made participants feel dismissed and decreased the likelihood of seeking medical care for more serious issues. Participants felt similarly dismissed when doctors did not maintain eye contact with them during the appointment. Doctors were often on the computer, and participants interpreted this both as a lack of knowledge and disinterest in their care: “*the doctors in here and when you tell them about your pain they open the internet or open the book in front of you*” (R5, M), *“you actually feel like they are busier with their stuff. And actually not treating the patients”* (R3, M). Participants described this as a key feature distinguishing between doctors they trust and ones they do not.

#### Language

While most participants were able to converse in English and indicated that they now spoke Norwegian, many discussed prior or current language barriers. Interpreters were not always reliable, and once participants learned Norwegian, many felt a new sense of autonomy and control over their healthcare experiences: “*when I speak to them in Norwegian I am by myself so I know how to experience it and I know how to tell them what I feel and where I feel it. I can say exactly what I feel. I like to express myself in Norwegian and not when the interpreter does it for me*” (R2, M). Participants specifically mentioned wanting more language training during their early days in Norway while living in reception centers.

#### Physician ethnicity

Issues of race and differences in sociocultural backgrounds also complicated interactions with doctors. Multiple participants described better healthcare interactions with doctors who were perceived as also not Norwegian: *“If they are from other countries, foreign countries, they are willing to help you. So I was lucky I was having my doctor he is from Pakistan. And the dentist for my daughter...she is from Somalia so they were good for us”* (R10, M). The same participant, however, was also concerned that there were so many foreign doctors in the Norwegian health system. The fact that there were so many foreigners to him meant that Norway did not do a good job of training their own doctors. This preference for foreign doctors may then be due to both perceived better care from someone who may have overlapping immigration experiences, and assumed issues with medical education in Norway.

However, other participants indicated a difference in their care based on the fact that they were not Norwegian. Participants described Norwegian-born friends receiving more details from their doctor about treatments, and having seen doctors who have been dismissive towards them act much friendlier with Norwegian patients, theoretically *“because some of them don’t like non-Norwegians or Africans*” (R7, F).

## Discussion

This study illuminates some of the challenges Ethiopian refugees and asylum seekers face in coming to Norway and accessing the healthcare system. Access has been described, by Penchansky and Thomas, to be composed of five dimensions that represent the degree of “fit” between consumer and system: availability, accessibility, accommodation, affordability, and acceptability [33]. Availability, the relationship between supply and demand for specific services, accommodation, the relationship between the supply system organization and the ability of the consumer to adapt to this organization, and acceptability, the relationship between consumers’ attitudes about characteristics of providers and medical practice and the actual characteristics of existing providers, are the dimensions most contested by our participants. These are discussed in detail below.

### Availability

Despite having full legal access to healthcare services upon arrival in Norway, participants did not perceive that they had the same rights as other residents of Norway while living in reception centers. This may have been due to the onsite health services that some reception centers provide, and the reliance on reception center workers to facilitate access to other services.

A recent report on transit centers also suggests that some asylum seekers perceive limited access to health services, particularly secondary services [30]. This may also reflect limited service capacity or reluctance to initiate healthcare until patients are in a stable living situation to ensure continuity of care. It may also be related to participants’ perception of residency papers as both a gateway to rights and a sign of recognition and legitimacy in the Norwegian context. Prior studies have shown that residency papers can hold such symbolic meaning [34]. Thus, newly arrived asylum seekers should be better informed of their rights to health services to improve their knowledge and understanding of the system.

### Accommodation

Our study shows that the uncertainty inherent in the asylum seeking process takes a significant toll on those who seek protection in Norway and may limit their ability to adapt to a new healthcare system. While participants were provided information about the healthcare system at various time points, most reported that this information was difficult to use, and that this made it difficult to understand the organization of Norway’s healthcare system. For example, a majority of the written materials given during the introductory program are provided in Norwegian [35, 36]. This information is therefore not accessible to asylum seekers who have not yet learned the language. Making appointments, how to call a doctor’s office, the availability of health services in preferred languages, and how long it takes to see a specialist were all topics that the participants thought were crucial for newly arrived asylum seekers to learn about. Information that is not actionable is of little use to asylum seekers and may play a role in delayed access. Health navigator/mediator interventions for specific health services and different groups have also shown promising results internationally [37–39]. Future studies should consider the success of such interventions that aim to improve knowledge about general health services in Norway.

One consequence of this difficulty accommodating to a new system is the decline in mental health that participants attributed to the lack of structure and helplessness of seeking asylum. Length of stay in reception centers has long been recognized as a risk factor for poor mental health outcomes in asylum seeking populations [40]. Although the range and severity of mental health problems may be related to differences in kinds of reception centers, the process of asylum seeking alone has been shown to have an association with a range of psychological disorders in children [41–47]. In line with other research, our study shows that such mental health difficulties can be expected in adult asylum seekers as well [48, 49]. Additionally, although participants were adults at the time of the interview, many entered Norway during their adolescence and therefore may have carried the psychological stress of resettlement with them since childhood. We found that the mental burden of the asylum-seeking process also affected how participants prioritized their own health.

Additional services targeting mental health and opportunities for work or study may provide substantial incremental benefit. While some reception centers in Norway have piloted interventions aimed at preventing mental health problems, and some offer various activities or opportunities for learning, there appears to be no standard protocol. Given the documented negative mental health effects of prolonged periods in asylum, strategies for improving the well-being of residents and minimizing stress levels should be prioritized in reception centers.

Further, the social dimensions of health and well-being have been shown to have a large impact on asylum seekers and refugees. Lintner and Elsen, in their assessment of the subjective well-being of asylum seekers in South Tyrol, Italy, posit that a sense of connectedness and belonging, understood as feeling oneself as part of a social system in which one is actively and meaningfully engaged and valued by others, is essential to a holistic approach to the health, well-being, and adaptation to this community [50]. Our results echo this sentiment, with feelings of uselessness and a lack of connectedness representing a major complaint from our participants. For our participants, this was mediated in large part by gaining residency status and emphasizes the need to consider the health of this population from a more holistic approach that includes a social dimension.

### Acceptability

Perceptions of provider attitudes and beliefs played a large role in participants’ healthcare experiences. The doctor-patient relationship was crucial to participants, and many stressed the importance of feeling valued in a healthcare interaction. This sentiment is supported by prior studies. As previously mentioned, a study of Somali refugees’ expectations of healthcare in Sweden found that unfulfilled expectations of medical encounters can result in disappointment which entails “a lack of trust and feelings of rejection” [16]. This can further complicate the barriers preventing resettled refugees from obtaining health services and seeking future healthcare. This research also suggested that such populations want doctors to know what’s wrong with them without being asked too many questions and without consulting a reference book (16). Such practices on the part of the physician fostered mistrust of the subsequent advice and diagnosis. For refugees who are accustomed to hearing a diagnosis and receiving treatment immediately, waiting for lab results or going to a doctor’s appointment in order to get a referral for an x-ray can be confusing [51].

The common suggestion from doctors to “just drink water” also echoes findings from Sweden. Somali respondents often indicated that upon visiting a doctor with a complaint they were told, ‘it’s really nothing’. This was perceived as a rejection and an uninterested response [16]. This response may well have been intended as reassurance of nothing serious, rather than a dismissal, but different interpretations due to different cultural backgrounds make such situations hard to navigate. Culturally based differences in expectations of healthcare interactions and in prescription practices may have also contributed to dissatisfaction. Dismissiveness may have also played a role in the feelings of discrimination that participants described. It is hard to determine whether perceptions of discrimination and dismissiveness originate in prejudice against immigrants or differences in communication style.

Participants felt similarly dismissed when having to wait long periods for a pre-arranged appointment and when their doctors did not look at them. In today’s era of electronic medical records (EMR), doctors often must sit facing a computer to take notes while their patients are talking. Dissatisfaction with this arrangement appears to cross cultural and geographic boundaries. Fortunately, however, there are ways to improve the interaction despite the requirements of the EMR.

Although anyone may feel resentment or mistrust in reaction to feeling dismissed by his physician, the likelihood and extent of such a perception may be heightened by differences in cultural background and language. Moreover, research in the U.S. has shown that physicians feel a specific inability to provide cross cultural care, in particular to new immigrants [52]. Similar sentiments were echoed by providers in Norway [20]. Interpersonal complications in healthcare interactions are pervasive, occur across cultural and geographic boundaries, and represent significant issues with healthcare delivery. However, interpersonal complications occur to a greater degree, and have more severe health seeking consequences, in populations with low health literacy and unfamiliarity with the language and customs of a new country. Training in cross-cultural competency should be a mandatory part of education for new healthcare professionals, and a part of continuing development for those already in the field.

### Strengths and limitations

Limitations of the study include the size of the sample and transferability of the findings. Although we interviewed a small number of participants, data saturation was achieved, with many participants echoing similar ideas. Data rigor was verified by comparing coding between the first and last author, and by the fact that many barriers discussed were in line with previous literature. Further, all themes were discussed with the study team and information from participants was cross-checked when contradictions occurred. Additionally, since participants were recruited from community contacts and subsequent snowball sampling, many participants came from similar social groups. For example, a large portion of participants were university students in their early 20s. It may be that being in such similar social groups increased data saturation artificially. Additionally, this cohort may represent a particularly resourceful group of people. This group also reported better health status than is the norm for refugee communities. It is reasonable to expect that difficulties in accessing health services would be more significant in older asylum seekers with less education or resources, or those with worse health status.

Our participants also resettled 3–10 years prior to the interview, and therefore needed to recall events from the past. While most similar research relied on populations of recently resettled refugees, our population is unique given that they were able to compare experiences over time, and had had time to reflect on the impact of these experiences. Due to the small sample size and limited variability between participants, we cannot be certain that these experiences are transferable to immigrants with different sociocultural characteristics from our sample. Additionally, since we interviewed only asylum seekers and no healthcare providers, we cannot comment on health care providers’ perspectives and how their knowledge of the asylum seeking process may have impacted healthcare experiences for this population.

An additional concern is the role of the researcher in data collection and analysis. The first author, an American woman living in Norway, conducted the interviews. Although conducting interviews in English could introduce a selection bias, only three participants chose to use an interpreter, although all were offered this service, and English is the most commonly spoken foreign language in Ethiopia. The first author also shared some experiences of living in a new country with the participants which may have made participants more comfortable speaking with her. Similarly, the university aged students were close in age and profession, and this may have also helped these participants feel comfortable. On the other hand, differences in ethnicity, sociocultural differences, and the power dynamic inherent in doing research may have introduced some distance or discomfort. Her experiences with the U.S. healthcare system, both as a user and as a medical student, and with refugees in the U.S. may have also influenced the analysis.

## Conclusion

The interviews shed light on healthcare experiences and barriers to care that Ethiopian refugees and asylum seekers face in Norway, and the ways in which these barriers are shaped by past experiences, expectations, and the social, cultural, and bureaucratic realities of life in Norway. The fact that this community was satisfied with many aspects of its health-related experiences speaks to the many successes of resettlement support agencies and local and national governments. There are still, however, areas that should be improved. Although some resettlement challenges are specific to country and population, many similarities exist between our participants and studies undertaken in other countries with different refugee communities. In this international context our results suggest that a more holistic approach to the health and wellness of asylum seeking communities would reduce the health-related challenges of resettlement.

For Norway in particular, this study highlights the need for intervention during the asylum seeking process. While there is a clear need for governments to maintain organization in the asylum seeking process, mental health deteriorates due to the lack of work, education, and intellectual stimulation that occurs during the time of resettlement. If temporary work permits are not possible, perhaps access to skills workshops relevant to common professions would make asylum seekers feel more productive and improve mental health. Further, there is much that could be done to improve the ways in which Ethiopian refugees and asylum seekers use the healthcare system. Providing information in multiple languages, multiple forms, and at opportune times could go a long way to enable asylum seekers to act on the information they receive. Finally, this study suggests that there is a need for cross cultural education for physicians and healthcare workers. Simply understanding how asylum seekers perceive statements such as “just drink water” may make healthcare workers more sensitive to differences in interpersonal interactions that asylum seekers bring to an appointment.

## Supplementary information

**Additional file 1.** Interview Guide.

## Data Availability

The datasets generated during the current study are not publicly available due to the sensitive and personal nature of the information contained in the data. Data may be available from the current authors, with restrictions and following ethical approval.

## Abbreviations

EMR: Electronic Medical Record
GP: General Practitioner
NAV: Norwegian Welfare and Labor Administration
NOK: Norwegian Krone
UDI: Norwegian Directorate of Immigration
UNHCR: United Nations High Commissioner for Refugees
