# Supplementary material for: A qualitative study of health experiences of Ethiopian asylum seekers in Norway
Source: BMC Health Serv Res. 2019 Dec 11;19:958. doi: 10.1186/s12913-019-4813-7 (PMC6907115; doi:10.1186/s12913-019-4813-7)
Supplement: Supplementary file 1 — Additional file 1. Interview Guide. [file 12913_2019_4813_MOESM1_ESM.docx]

**Additional file 1 - Interview Guide**

**Background Information**

1. Demographics:
   1. Age
   2. Gender
   3. What part of the country are you from?
   4. Length of time in Norway
   5. Immigrant status e.g. asylum seeker/refugee
   6. Employment status
   7. Education level – number of years in school?
2. Can you tell me about how you came to live in Norway? Why Norway?
   1. Where are you from? How/why did you come here?
   2. What is your living situation like now?
      1. Did you come with any family members? Who do you live with now?
3. How would you assess your current health -- very good, good, fair, poor, very poor?

**General Health Experiences**

1. Do you remember if you were offered the routine health consultation for refugees and asylum seekers in your first few months in Norway?
   1. If yes, did you follow up on the offer? What was your experience with this like? How helpful did you find it?
   2. If you did not, is there a reason why you did not?
2. How have you found Norwegian health services so far?
   1. What has been your best/worst health experience here?
      1. Please explain
   2. How do you feel in a hospital or a doctor’s office in Norway? What makes you feel this way?
      1. E.g. comfortable, nervous, sad, uncomfortable, happy, etc.
   3. How do your experiences in Norway compare to experiences back home?
      1. Are there many differences? Is it easier to see a doctor here or at home?
   4. Do you have any chronic illnesses that have required medical care here?
      1. If yes, how would you describe that experience?
   5. Have you had any acute illnesses that have required medical care here?
      1. If yes, please describe those experiences.
   6. Do you know which vaccines you had in your home country?
      1. Have you had an assessment of your vaccination status after arrival in Norway?
      2. Have you received any vaccines since arriving in Norway?
   7. If you felt sick tomorrow, what would you do?

**Structural and Informational Barriers**

1. How have you received information about healthcare in Norway?
   1. Is there a specific person or organization that has been most helpful with this? In what ways was this person (or other source) most helpful?
   2. Is there any other information you would have liked to have received? What would have made using the Norwegian health system easier for you?
2. How would you describe the costs associated with the Norwegian healthcare system – too expensive/very cheap, etc.
   1. Has payment ever been difficult for you?
3. Do you feel like you understand the Norwegian healthcare system?
4. How would you describe it to a friend?
5. Have you visited your general practitioner?
   - 1. If yes: How do you find the appointment system? (making appointment, waiting times, enough time with doctor getting to and from etc). How did you know who your doctor was?
     2. Would you know what to do if you wanted to change doctors?
     3. How do you find the referral system? (gatekeeper vs. direct secondary services)
     4. If no: do you know who your GP is, how to make an appointment etc
6. Have you ever consulted with a specialist?
   1. Have you ever had difficulty finding a particular type of doctor? Ex. Dentists, eye doctors, or surgeons
   2. Were you referred or did you have to find the doctor on your own?
7. What kinds of health centers have you used here?
8. E.g. out of hours (legevakt), hospital, community health centre (helsestasjon)?
9. Any notable experiences here?

**Cultural and Linguistic Barriers**

1. Have you felt like your health care providers are sensitive/understanding of the way you perceive your health problems/issues? Explain how so or how not.
2. Does he/she listen to your problems?
3. How do you feel about the care your doctor is providing?
4. Do you trust your doctor? (confidentiality, expertise, etc.)
5. How do you communicate with health professionals?
6. If uses an interpreter: how do you feel about using an interpreter? What do you feel are the advantages / disadvantages with using an interpreter?
7. have you used both interpreters in person and on the phone? Is there a method you prefer and why?
8. How does the gender or cultural background of your interpreter influence your comfort level?
9. If speaks alone: Have you felt that you and your doctor understand each other sufficiently?
10. If no: why do you not use interpretive services?
11. What are your expectations of a healthcare interaction?
    1. Do you find that these expectations are met/unmet?

**Final**

1. Is there anything else about your healthcare experience in Norway that you would like to add?
2. What do you think could be done to improve healthcare experiences in Norway?
